# Quality assessment and consumer acceptability of bread from wheat and fermented banana flour

**DOI:** 10.1002/fsn3.298

**Published:** 2015-10-17

**Authors:** Abiodun Omowonuola Adebayo‐Oyetoro, Oladeinde Olatunde Ogundipe, Kehinde Nojeemdeen Adeeko

**Affiliations:** ^1^ Department of Food TechnologyYaba College of TechnologyP.M.B 2011YabaLagosNigeria

**Keywords:** Acceptability, banana, fermented, quality, wheat

## Abstract

Bread was produced from wheat flour and fermented unripe banana using the straight dough method. Matured unripe banana was peeled, sliced, steam blanched, dried and milled, and sieved to obtain flour. The flour was mixed with water and made into slurry and allowed to stand for 24 h after which it was divided into several portions and blended with wheat flour in different ratios. Proximate and mineral compositions as well as functional, pasting, and sensory characteristics of the samples were determined. The results of proximate analysis showed that crude fiber ranged between 1.95% and 3.19%, carbohydrate was between 49.70% and 52.98% and protein was 6.92% and 10.25%, respectively, while iron was between 27.07 mg/100 g and 29.30 mg/100 g. Swelling capacity of the experimental samples showed a significant difference from that of control. Peak viscosity ranged between 97.00RVU and 153.63RVU for experimental samples compared with 392.35RVU obtained for the control. Most of the sensory properties for the experimental samples were significantly different from the control. This study showed that bread with better quality and acceptability can be produced from wheat–unripe banana blends.

## Introduction

Bread is the loaf that results from the baking of dough which is obtained from a mixture of flour, salt, sugar, yeast, and water. Other ingredients like fat, milk, milk solids, egg, anti‐oxidants, etc. may be added. Bread is an important food whose consumption is steady and increasing in Nigeria. It is, however, relatively expensive because it is made from wheat flour which has to be imported (Edema et al. 2004). Bread is an important staple food both in the developed and developing world (Abdelghafor et al. 2011). In India, bread has become one of the most widely consumed nonindigenous food (Das et al. 2012), whereas in Nigeria, bread has become the second most widely consumed nonindigenous food after rice (Shittu et al. 2007). Efforts have been made to promote the use of composite flour, in which flours can be made from locally grown crops replace a portion of wheat for use in bread, thereby, decreasing the demand for imported wheat and producing protein enriched bread (Giami et al. 2005). The predominance of wheat flour for baking aerated breads is due to the properties of its elastic gluten protein which helps in producing a relatively large loaf volume with a regular, finely vesiculated crumb structure.

Banana (*Musa* spp) constitutes a rich energy source with carbohydrate accounting for 22–32% of the fruit weight. It is rich in vitamins A, B_6,_ and C as well as minerals particularly potassium, magnesium, phosphorus, and folate (Chandler 1995; Honfo et al. 2007a,b). Banana is a major staple crop for millions in developing countries. Meanwhile, a lot of postharvest losses result from the large quantities of this crop produced annually. It is therefore imperative to introduce diversification to its use thereby reducing this wastage. Preparation of banana flour from unripe flour has been reported by some researchers (Rodriguez‐Ambriz et al. 2008). Acceptable bread from wheat–plantain composite flour using up to 80:20 w/w ratios of wheat: mature green plantain flour has been reported (Mepba et al. 2007). The aim of this study is to produce and evaluate the quality of bread prepared from blends of wheat and banana flour.

## Materials and Methods

### Materials

Hard wheat flour, bakers’ yeast, granulated sugar, table salt, baking fat, and vegetable fat were purchased from Ojuwoye market, Mushin, Lagos. The unripe banana bunch was purchased from the fruit market, Ketu. Production of bread was carried out in the laboratory of the department of Food Technology, Yaba College of Technology, Yaba, Lagos, Nigeria.

### Sample preparation

**Figure 1:**
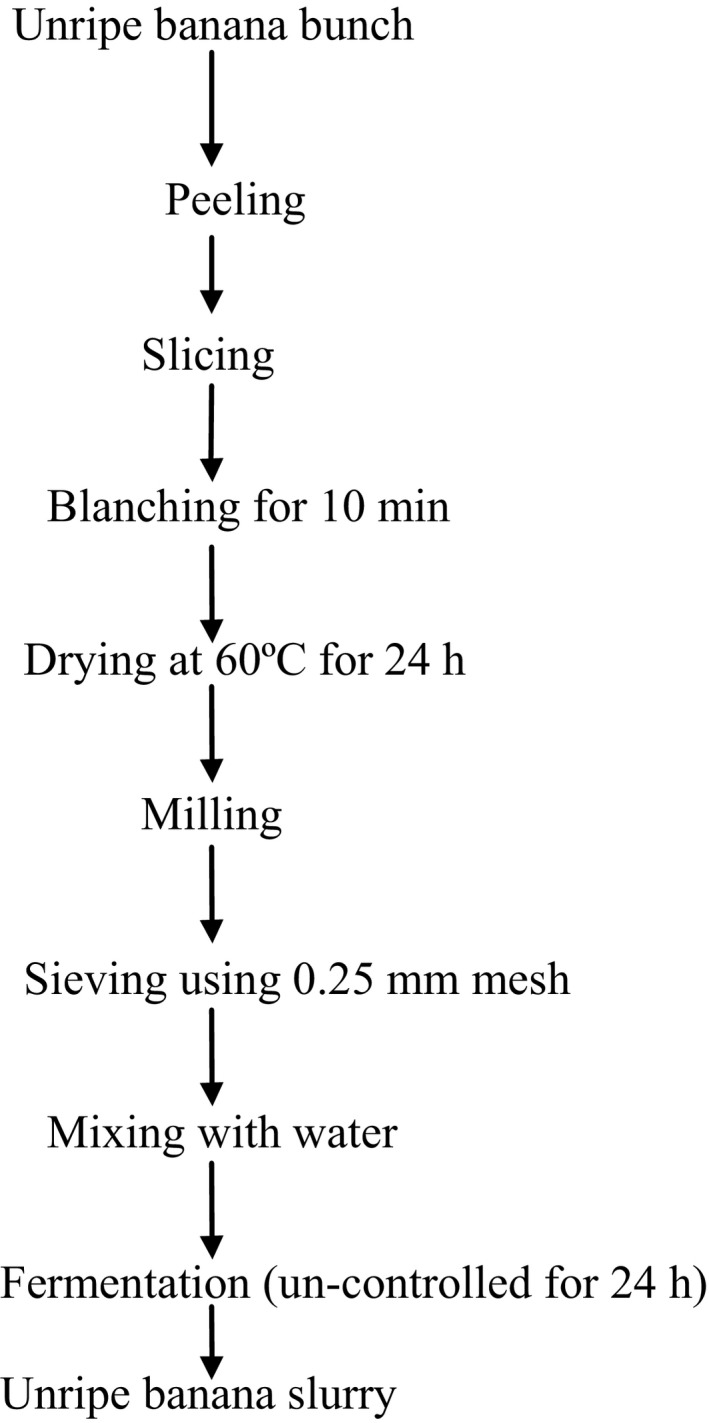
Flowchart for the production of unripe banana slurry. Source: Modified method of Oloyede et al. (2013).

The banana slurry was produced by the modified method of Oloyede et al. (2013). A bunch of unripe banana whose peel is green and whose pulp is not soft was washed, peeled, and cut into round slices of 10 mm thickness. The slices obtained were steam blanched for 10 min, dried at 60°C for 24 h, milled, sieved, and mixed with water (10 g flour/3 mL water) before fermentation for 24 h. The fermented slurry was now mixed with wheat flour in ratio 90:10, 80:20, and 70:30 and other ingredients such as fat, yeast, and salt to make bread (Fig. 1).

### Formulation of blends

Composite flour samples containing wheat and unripe banana flour were formulated, using ratio 100:0, 90:10, 80:20, and 70:30 and coded CON, KAN, TAD, and OAV, respectively.

### Recipe for bread

Flour —————100%

Water—————65%

Fat——————3%

Sugar—————–6%

Salt and yeast————2–2.5% (Raymond, 2001 and Saus, 2008).

Fifty‐five percentage water was used in this study because the banana was in the slurry form instead of the dried powder.

### Production of bread using composite flour

**Figure 2:**
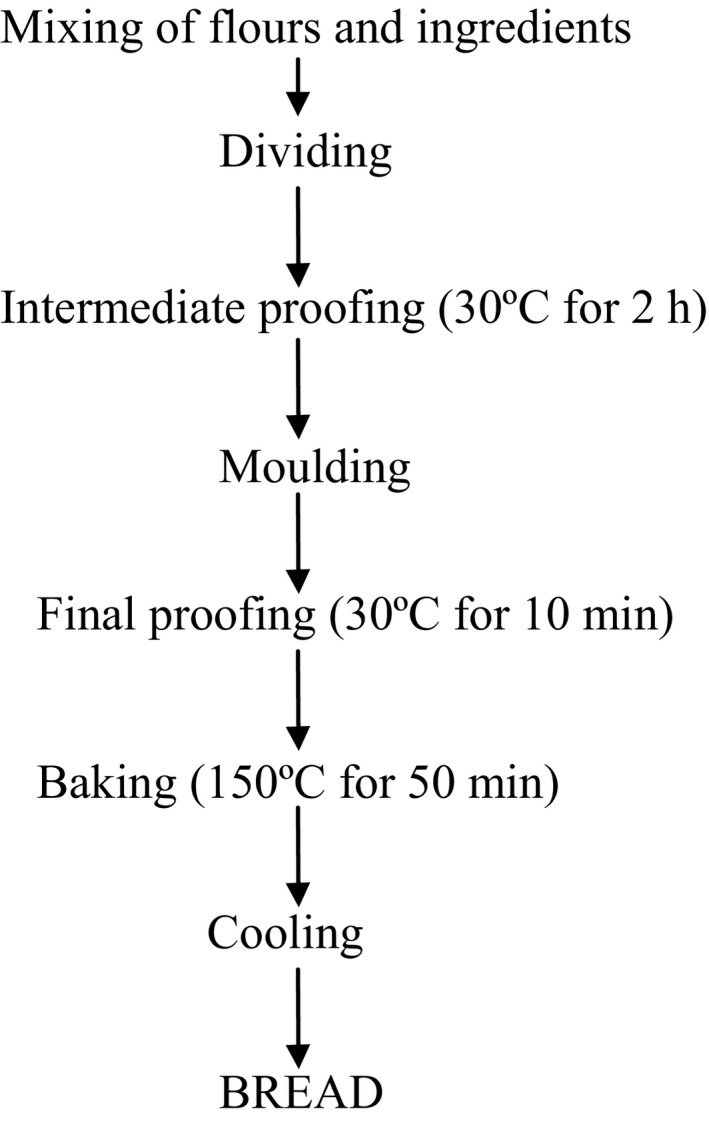
Flowchart for straight dough method of bread making. Source: Badifu and Akaa (2001).

The straight dough method of bread making as described by Badifu and Akaa (2001) was used (Fig. 2).

### Proximate composition

Proximate analyses of the bread samples were carried out using the AOAC (2005) for protein, crude fiber, fat content, ash content and moisture content. Carbohydrate content was determined by difference.

### Functional properties

Bulk density of the flour was determined using the method of Onwuka (2005) while the swelling capacity was determined according to the method described by Oladele and Aina (2007).

### Pasting properties

The pasting properties of samples were assessed in an RVA‐4 (Rapid Visco Analyzer), using the RVA general pasting method.

### Sensory evaluation

The sensory analysis was carried out using the scoring test as described by Akinjayeju (2009). The sensory attributes of the bread samples including crust color, crumb color, texture, taste, and aroma were evaluated about 1 h after baking by a semitrained 15‐member panel that are familiar with bread.

### Statistical analysis

All analysis was conducted in duplicate and the data were all subjected to ANOVA (analysis of variance) as described by Akinjayeju (2009) while the mean will be separated by DMRT (Duncan Mean Range Test).

## Results and Discussion

Table 1 shows the proximate composition of wheat–plantain composite bread. Moisture content ranged between 28.94% and 36.95% with sample CON having the least and sample OAV having the highest, this was in agreement with the findings of Olaoye and Onilude (2011). It was observed that as the substitution level increases the moisture content also increases. Highest amount of ash is in sample OAV with 1.59 ± 0.09% and the least was in sample KAN 1.11 ± 0.01%. This was in agreement with the findings of (Mongi et al. 2011). It was also observed that the crude protein was very high in CON with a value of 10.25 ± 0.01%, followed by KAN, TAD, and OAV with 9.41 ± 0.01%, 8.30 ± 0.14%, and 6.92 ± 0.02%, respectively. Sample CON has the highest value of carbohydrate with mean score of 52.98 ± 0.01. It was observed that the carbohydrate content was reducing with increase in the substitution level. This is in agreement with Olaoye and Onilude (2011) and contrary to the findings of (Mepba et al. 2007). The results showed significant difference between the proximate compositions of the samples.

**Table 1:** Proximate composition of bread samples

Sample	Moisture (%)	Ash (%)	Fat (%)	Crude fiber (%)	Crude protein (%)	Carbohydrate
CON	28.94 ± 7.32^c^	0.93 ± 0.04^b^	1.81 ± 0.00^a^	0.05 ± 0.01^d^	10.25 ± 0.13^a^	52.98 ± 0.01^a^
KAN	34.71 ± 0.27^b^	1.11 ± 0.01^a^	1.76 ± 0.00^a^	1.95 ± 0.52^c^	9.41 ± 0.01^b^	51.09 ± 0.00^a^
TAD	35.26 ± 0.00^b^	1.49 ± 0.05^a^	1.72 ± 0.04^a^	2.44 ± 0.62^b^	8.30 ± 0.14^b^	50.85 ± 0.05^a^
OAV	36.95 ± 0.21^a^	1.59 ± 0.09^a^	1.66 ± 0.03^a^	3.19 ± 0.13^a^	6.92 ± 0.02^c^	49.70 ± 0.15^b^

Sample CON: 100% wheat flour, KAN: 90% wheat and 10% unripe banana flour, TAD: 80% wheat and 20% unripe banana flour, OAV: 70% wheat and 30% unripe banana flour.

Mean values with the same letter within the same column are not significantly (*P* > 0.05) different.

Table 2 shows the mineral composition of flour samples. Minerals are essential nutrients that are needed in the body to facilitate proper functioning of certain organs (Amoakoah et al. 2015). Some minerals are needed in smaller quantity (micro) while others are needed in larger quantity (macro). This study analyzed iron and zinc as macro and micro nutrients, respectively. The result showed that the concentration of iron (Fe) in sample OAV is higher than other two samples with a mean value of 30.09 mg/100. Meanwhile the least concentration of iron (Fe) is found in the experimental sample KAN with value of 29.00 mg/100. However, the result also showed that the amount of iron in sample CON (27.07 mg/100) is lower than the other samples which were KAN (29.00 mg/100), TAD (29.39 mg/100), and OAV (30.09 mg/100), respectively. Furthermore, the table showed that the highest amount of Zinc (Zn) was found in sample OAV with a mean value of 31.36 mg/100, while sample KAN has the lowest value of 30.24 mg/100. It was observed that there is a significant difference between the experimental samples and the control samples in terms of iron and zinc content.

**Table 2:** Mineral composition of wheat/plantain flour blend

Samples	Iron (Fe) mg/100 g	Zinc (Zn) mg/100 g
CON	27.07 ± 1.17^b^	29.19 ± 1.31^b^
KAN	29.00 ± 0.17^a^	30.24 ± 0.08^a^
TAD	29.39 ± 0.18^a^	30.61 ± 0.38^a^
OAV	30.09 ± 0.22^a^	31.36 ± 0.16^a^

Sample CON: 100% wheat flour, KAN: 90% wheat and 10% unripe banana flour, TAD: 80% wheat and 20% unripe banana flour, OAV: 70% wheat and 30% unripe banana flour.

Mean values with the different letters within the same column are not significantly different at (*P* > 0.05).

Table 3 shows the functional properties of wheat–plantain flour blends. The bulk density ranged from 0.72 to 0.78 g/cm^3^ with sample OAV having the highest value while sample TAD has the lowest amount. This means the higher the bulk density, the denser the flour (Eke‐Ejiofor and Kiin‐Kabari 2012), suggesting that sample TAD is denser than other substituted samples. There is no significant difference between the control sample and samples KAN AND TAD. Swelling capacity is regarded as the quality criterion in some good formulations such as bakery products (Osungbaro et al. 2010). The sample OAV exhibited highest the swelling power 64% while sample KAN exhibited the lowest swelling power with 40%. Fermentation of the unripe banana flour and substitution level was observed to influence progressive increase in the swelling capacity. Fermentation and sun drying had been observed to clearly play a role in obtaining starch with high swelling power and desirable organoleptic properties. This has been found to facilitate production of high‐quality cassava‐wheat composite flours of which demand exist in bread making and various confectionery industries (Duffour et al. 1996). Sample OAV was significantly different from other samples in terms of bulk density while a significant difference also existed between samples CON and KAN, and other samples (TAD and OAV), respectively.

**Table 3:** Functional properties of wheat/plantain flour blend

Samples	Bulk density (%)	Swelling capacity (%)
CON	0.75 ± 0.17^a^	40 ± 0.00^c^
KAN	0.78 ± 0.01^a^	40 ± 0.00^c^
TAD	0.79 ± 0.03^a^	53 ± 1.29^b^
OAV	0.72 ± 0.14^b^	64 ± 2.71^a^

Sample CON: 100% wheat flour, KAN: 90% wheat and 10% unripe banana flour, TAD: 80% wheat and 20% unripe banana flour, OAV: 70% wheat and 30% unripe banana flour. Mean values with the different letters within the same column are not significantly different at (*P* > 0.05).

The pasting properties of the flour samples are shown in table 4. The pasting properties of starch are used in assessing the suitability of its application as functional ingredient in food and other industrial products (Oluwalana and Oluwamukomi 2011). The most important pasting characteristics is its amylographic viscosity (Sandhu et al. 2007). The pasting temperature of the flour samples ranges between 76.60°C and 87.57°C and the control sample is 87.2°C. The pasting temperature is a measure of the minimum temperature required to cook a giving food sample (Sandhu et al. 2007). The peak time is the measure of the cooking time (Adebowale et al. 2005). Both KAN and CON have the highest peak time of 5.97 min and 5.97 min, respectively, followed by TAD with 5.87 min while OAV has the lowest value of 5.70 min. Peak viscosity is often correlated with the final product quality. It also provides an indication of the viscous load likely to be encountered during mixing (Maziya‐Dixon et al. 2004). Higher swelling capacity is indicative of higher peak viscosity while higher solubility as a result of starch degradation or dextrinization results in reduced paste viscosity (Shittu et al. 2001). The hold period sometimes called shear thinning, holding strength, hot paste viscosity, or trough due to the accompanied breakdown in viscosity is a period when the sample was subjected to a period of constant temperature (usually 95°C) and mechanical shear stress. It is the minimum viscosity value in the constant temperature phase of the RVA profile and measures the ability of paste to withstand breakdown during cooling (Newport, 1998). Sample KAN has the highest trough value 115.8RVU followed by TAD with a value of 110.6RVU and the least was OAV with 101.2RVU, while the control sample has a trough value of 224.75 RVU. This period is often associated with a breakdown in viscosity (Ragaee et al. 2006). It is an indication of breakdown or stability of the starch gel during cooking. The lower the value the more stable is the starch gel. The breakdown is regarded as a measure of the degree of disintegration of granules or paste stability (Newport Scientific 1998). The breakdown viscosities ranged between 52.45RVU and 81.2RVU and the control sample CON had the highest break down viscosity value of 224.75RVU. The viscosity after cooling to 50°C represents the setback or viscosity of cooked paste. It is a stage where retrogradation or reordering of starch molecules occurs. Higher setback values are synonymous to reduced dough digestibility (Shittu et al. 2001), while lower setback during cooling of the paste indicates lower tendency for retrogradation (Izonfuo and Omuaru 1988). The final viscosities of the samples were KAN (227.95RVU), TAD (215.5RVU), OAV (191.85RVU), and 384.20RVU for CON. The setback value for the control sample CON was 159.45RVU, while KAN, TAD, and OAV have 227.95RVU, 215.5RVU, and 191.85RVU, respectively. The setback viscosity indicates the tendency of the dough to undergo retrogradation, a phenomenon that causes dough to become firmer and increasingly resistant to enzyme attack (Ihekoronye and Ngoddy 1985). This has a serious implication on the digestibility of the dough when consumed. Higher setback values are synonymous to reduced dough digestibility (Shittu et al. 2001), while lower setback during the cooling of the paste indicates lower tendency for retrogradation (Sandhu et al. 2007).

**Table 4:** Pasting properties of samples

Samples	Peak (RVU)	Trough 1 (RVU)	Breakdown viscosity (RVU)	Final viscosity (RVU)	Setback (RVU)	Time (min)	Pasting temp. (°C)
KAN	197.00 ± 2.53	115.80 ± 18.38	81.25 ± 0.91	227.95 ± 1.85	112.15 ± 3.23	5.97 ± 0.05	87.57 ± 0.60
TAD	183.95 ± 1.85	110.60 ± 11.31	73.35 ± 3.53	215.50 ± 2.28	104.90 ± 1.97	5.87 ± 0.09	87.20 ± 0.07
OAV	153.65 ± 1.77	101.20 ± 5.65	52.45 ± 2.12	191.85 ± 1.61	90.65 ± 0.95	5.70 ± 0.05	86.83 ± 0.53
CON	392.35 ± 2.15	224.75 ± 12.36	167.60 ± 1.79	384.20 ± 1.98	159.45 ± 0.63	5.97 ± 0.33	76.60 ± 0.85

Sample CON: 100% wheat flour, KAN: 90% wheat and 10% unripe banana flour, TAD: 80% wheat and 20% unripe banana flour, OAV: 70% wheat and 30% unripe banana flour.

The Table 5 below shows the result of sensory analysis. The result showed that sample KAN is the most acceptable in terms of crumb and crust color with mean score of 4.5 and 6.0 while sample OAV is the least preferred in terms of crumb and crust color, respectively. As the level of substitution increases, the acceptance of crumb color decreases. With reference to taste and aroma, sample KAN is the most preferred with an average mean scores of 5.5 and 5.0, respectively followed by sample TAD with mean scores of 4.0 and 3.5 while sample OAV is the least accepted with mean scores of 2.5 and 3.0, respectively. Sample KAN was the most preferred in terms of overall acceptability with an average mean score of 5.0 while sample OAV is the least accepted with a score of 2.5. This is as a result of substitution of wheat flour with unripe banana flour. These results are similar to the findings of Mongi et al. (2011) and Mepba et al. (2007). It was also observed that a significant difference occurs between the control sample and most of the experimental samples except KAN in most of the sensory parameters measured.

**Table 5:** Sensory analysis of bread samples

Samples	Crumb color	Crumb texture	Crust color	Taste	Aroma	Overall acceptability
CON	5.0^a^	5.5^a^	6.2^a^	5.8^a^	5.2^a^	5.5^a^
KAN	4.5^b^	5.0^a^	6.0^a^	5.5^a^	5.0^a^	5.0^a^
TAD	4.0^b^	4.0^b^	3.5^b^	4.0^b^	3.5^a^	3.5^b^
OAV	3.0^c^	2.5^c^	3.5^b^	2.5^c^	3.0^a^	2.5^c^

KAN: 90% wheat and 10% unripe banana flour, TAD: 80% wheat and 20% unripe banana flour, OAV: 70% wheat and 30% unripe banana flour.

Mean values with the different letters within the same column are not significantly different at (*P* > 0.05).

## Conclusion

Addition of unripe banana was found to increase the crude fiber, ash, iron, and zinc content of the bread samples. Meanwhile, the products were acceptable by the sensory panelists although a significant difference was observed between the control and the experimental samples used in this study.

## Conflict of Interest

Authors hereby declare that no conflict of interest exists.
